# In this month’s Bulletin

**DOI:** 10.2471/BLT.25.000525

**Published:** 2025-05-01

In the editorial section, Takahiro Nanri (286) commemorates 50 years of a WHO partnership on leprosy elimination. José Ramos-Horta et al. (287) detail commitments to climate and health goals in Timor-Leste. 

Gary Humphreys (290–291) reports on the public health intersection of building standards and urban fires. Susana Vázquez Torres talks to Ana Lesher-Treviño (292–293) about the potential for computational protein design to improve snake antivenoms.

## Egypt

### Improving central line management in neonatal intensive care

Hoda Mohamed Owais et al. (343–348) track efforts to reduce infections.

## India

### Updating the National Cancer Grid

C S Pramesh et al. (337–342) describe the introduction of electronic medical records.

## Malawi

### HIV, malaria and tuberculosis

Tara Danielle Mangal et al. (304–315) model outcomes of a decade of disease prevention and control initiatives.

## Global

### City dwellers and extreme weather

Mary Catherine Sheehan et al. (294–303) assess use of early warning systems.

### Treatment for late syphilis

Rochelle Tobin et al. (316–327) identify factors influencing treatment completion rate. 

### Food crops and public health

Erica Reeve et al. (328–336) summarize arguments for changing agricultural subsidies.

